# A Novel Toxin from *Haplopelma lividum* Selectively Inhibits the Na_V_1.8 Channel and Possesses Potent Analgesic Efficacy

**DOI:** 10.3390/toxins9010007

**Published:** 2016-12-26

**Authors:** Ping Meng, Honggang Huang, Gan Wang, Shilong Yang, Qiuming Lu, Jingze Liu, Ren Lai, Mingqiang Rong

**Affiliations:** 1 Key Laboratory of Animal Models and Human Disease Mechanisms of Chinese Academy of Sciences & Yunnan Province, Kunming Institute of Zoology, Kunming 650223, Yunnan, China; mengping@mail.kiz.ac.cn (P.M.); yslzoology@163.com (S.Y.); lvqm@mail.kiz.ac.cn (Q.L.); 2 United Laboratory of Natural Peptide of University of Science and Technology of China & Kunming Institute of Zoology, Chinese Academy of Science, Kunming 650223, Yunnan, China; 3 Sino-African Joint Research Center, Chinese Academy of Science, Wuhan 430074, Hubei, China; 4 Department of Biochemistry and Molecular Biology, University of Southern Denmark, Odense 5230, Denmark; hhuang@bmb.sdu.dk; 5 The Danish Diabetes Academy, Odense 5230, Denmark; 6 Life Sciences College, Nanjing Agricultural University, Nanjing 210095, Jiangsu, China; wang.gan@outlook.com; 7 Key Laboratory of Animal Physiology, Biochemistry and Molecular Biology of Hebei Province, College of Life Sciences, Hebei Normal University, Shijiazhuang 050024, Hebei, China

**Keywords:** spider, venom, neurotoxin, Na_V_1.8, analgesia

## Abstract

Spider venoms are a complex mixture of peptides with a large number of neurotoxins targeting ion channels. Although thousands of peptide toxins have been identified from venoms of numerous species of spiders, many unknown species urgently need to be investigated. In this study, a novel sodium channel inhibitor, µ-TRTX-Hl1a, was identified from the venom of *Haplopelma lividum*. It contained eight cysteines and formed a conserved cysteine pattern of ICK motif. µ-TRTX-Hl1a inhibited the TTX-resistant (TTX-r) sodium channel current rather than the TTX-sensitive (TTX-s) sodium channel current. Meanwhile, µ-TRTX-Hl1a selectively inhibited Na_V_1.8 with an IC_50_ value of 2.19 μM. Intraperitoneal injection of µ-TRTX-Hl1a dose-dependently reduced inflammatory and neuropathic pain in rodent models of formalin-induced paw licking, tail-flicking, acetic acid-induced writhing, and hot plate test. It showed a better analgesic effect than morphine in inflammatory pain and equipotent effect to morphine in neuropathic pain. These findings demonstrate that µ-TRTX-Hl1a might be a valuable tool for physiology studies on Na_V_1.8 and a promising lead molecule for pain therapeutics.

## 1. Introduction

About 46,340 species of spiders live in the world, but only 1407 spider toxins have been identified from the venom of 97 species, whereas venom peptides from other species of spiders are still unknown [1]. Numerous evidence demonstrates that spider venoms are complex chemical cocktails and the major components are small, disulfide-rich neurotoxins. Most of the neurotoxins are sodium and calcium channel modulators. They can cause instant paralysis of prey, which may be a very effective way for spiders to immobilize prey. The venom of *Haplopelma lividum* has not been well characterized yet [2]. A case report of a finger bite from *H. lividum* showed that the venom caused severe pain, implying that neurotoxins may exist in the venom [3].

Chronic pain is a major problem which plagues many people in the world and consequently causes a significant economic loss [4]. Although the mechanism of pain is not well understood, it is believed that voltage-gated sodium channels, including Na_V_1.8, play an important role. Voltage-gated sodium channels (VGSCs) are integral transmembrane proteins distributed widely in most excitable tissues and are essentially involved in the upstroke of action potentials [5,6]. They form the basis for electrical signaling in the nervous system and control the flow of ions across the plasma membrane in response to voltage changes [7]. So far, nine distinct subtypes of sodium channels (Na_V_1.1–Na_V_1.9) have been cloned from a mammal. The TTX-resistant (TTX-r) Na_V_1.8 channel is mainly expressed in, but not restricted to, the peripheral nervous system [8]. Na_V_1.8 mutations could produce peripheral mechanical hypersensitivity and hyperexcitability in rat dorsal root ganglion (DRG) neurons [9,10]. Methylglyoxal could induce post-translational modifications of Na_V_1.8 [11]. Hence, the electrical excitability and facilitated firing of nociceptive neurons were strongly increased to evoke thermal and mechanical hyperalgesia. A-803467, a Na_V_1.8 channel blocker, could reduce allodynia in spinal nerve ligation, sciatic nerve injury, capsaicin-induced secondary mechanical allodynia, and thermal hyperalgesia [12].

In this work, a novel neurotoxin (µ-TRTX-Hl1a) with 39 amino acid residues was identified from the venom of the spider *H. lividum* [13]. µ-TRTX-Hl1a showed a selective inhibition on the sodium channel subtype Na_V_1.8. Tests in several animal models (formalin-induced paw licking, tail-flicking test, hot plate test, and acetic acid-induced writhing) indicated that it has strong analgesic effects.

## 2. Results

### 2.1. Sequence Analysis of µ-TRTX-Hl1a

**Figure 1:**
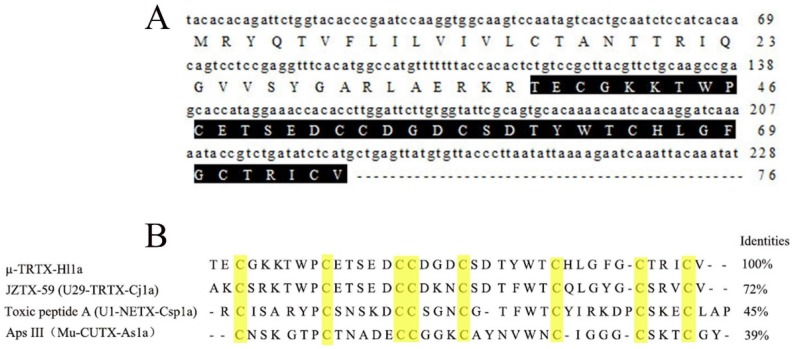
Amino acid and cDNA sequence of µ-TRTX-Hl1a. (**A**) The cDNA sequence of µ-TRTX-Hl1a. The sequence of the mature peptide is highlighted in black; and (**B**) alignment of µ-TRTX-Hl1a with related spider toxins from *Chilobrachys jingzhao* (JZTX-59), *Calisoga* sp. (Toxic peptide C (U1-NETX-Csp1a)), and *Apomastus schlingeri* (ApsIII (μ-CUTX-As1a)).

The original cDNA library of the *H. lividum* venom gland contained 1.5 × 10^6^ independent clones according to the protocol from cDNA library construction kit. To extract high-quality sequence, vectors, primer sequences, poly (A) tails, and sequences shorter than 200 bp were excluded. Finally, 381 EST sequences were generated by DNA sequencing. A peptide named µ-TRTX-Hl1a with eight cysteines formed a conserved cysteine pattern of ICK motif (Figure 1B). As shown in Figure 1, the full length of the cDNA open reading frame was 228 bp and encoded a 76-residue precursor peptide. The peptide consisted of a signal peptide of 19 residues, a mature peptide of 39 residues and an intervening propeptide of 18 residues. The mature peptide of µ-TRTX-Hl1a is TECGKKTWPCETSEDCCDGDCSDTYW TCHLGFGCTRICV. An online BLAST search showed that µ-TRTX-Hl1a had considerable sequence similarity with other spider toxins such as JZTX-59 (U29-TRTX-Cj1a), toxic peptide C (U1-NETX-Csp1a) and Aps III (μ-CUTX-As1a). Toxic peptide C and Aps III appear to be pore blockers and bind to the outer vestibule of insect voltage-gated sodium channels [14,15,16].

### 2.2. Synthesis and Refolding of µ-TRTX-Hl1a

**Figure 2:**
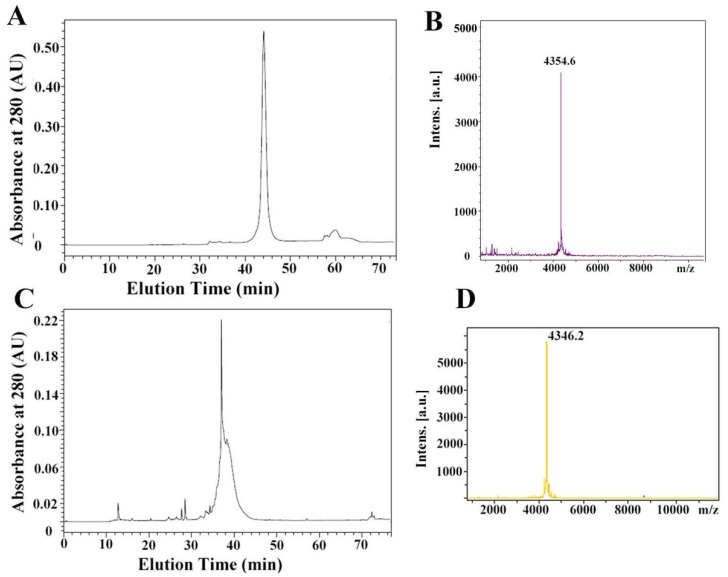
Identification of linear and refolded µ-TRTX-Hl1a; (**A**) the linear µ-TRTX-Hl1a was analyzed by C_18_ RP-HPLC. The elution was performed with the gradients of acetonitrile in 0.1% (*v*/*v*) trifluoroacetic acid in water at a flow rate of 1 mL/min. The gradient of acetonitrile was increased from 10% to 50% during the time of 10 to 50 min; (**B**) MALDI-TOF-MS determination of the molecular weight of linear µ-TRTX-Hl1a; (**C**) the refolded µ-TRTX-Hl1a was purified on C_18_ RP-HPLC. The elution was performed with the gradients of acetonitrile in 0.1% (*v*/*v*) trifluoroacetic acid in water at a flow rate of 1 mL/min. The gradient of acetonitrile was increased from 10% to 50% during the time of 10 to 50 min; (**D**) MALDI-TOF-MS determination of the molecular weight of refolded µ-TRTX-Hl1a.

In order to investigate its activity, µ-TRTX-Hl1a was synthesized as a linear peptide and its molecular weight was the same as the theoretical weight (4354.85 Da). Then, 0.5 mg of µ-TRTX-Hl1a was dissolved in different Tris-HCl buffers to test the folding yield to explore the best conditions for refolding (Table 1). The molecular weight of refolded peptide was 4346.2 Da (Figure 2D). The folding percentage was calculated by comparing areas of HPLC peaks for the linear and folded peptide. Firstly, 0.1 mM Tris-HCl buffer with 0.3 mM GSSG and 3 mM GSH was used as a folding buffer and the folding reaction was kept at different temperature for various duration. The highest folding percentage under those conditions was 8.52% (Table 1). 0.5 M *L*-Arg was introduced to Tris-HCl buffer in order to increase the folding rate. The folding percentages at pH 7.6, 7.4, 7.2, and 6.8 were 8.13%, 8.53%, 9.39%, and 10.38%, respectively. The folding percentage in the buffer (50 mM Tris-HCl, 50 mM NaCl, 0.15 mM GSSG, 1.5 mM GSH, 0.5 M *L*-Arg) at pH 6.8 and 7.2 was 10.20% and 13.46%, respectively.

As shown in Figure 2, the retention time of the linear peptide was 42.1 min with an average molecular mass of 4354.6 Da, while the folded peptide was eluted at 38.6 min and had a molecular mass of 4346.2 Da. The fact that the molecular mass of the refolded peptide was 8 Da less than that of the linear form indicates that four disulfide bonds were formed.

### 2.3. The Secondary Structure and Disulfide Bond of µ-TRTX-Hl1a

**Figure 3:**
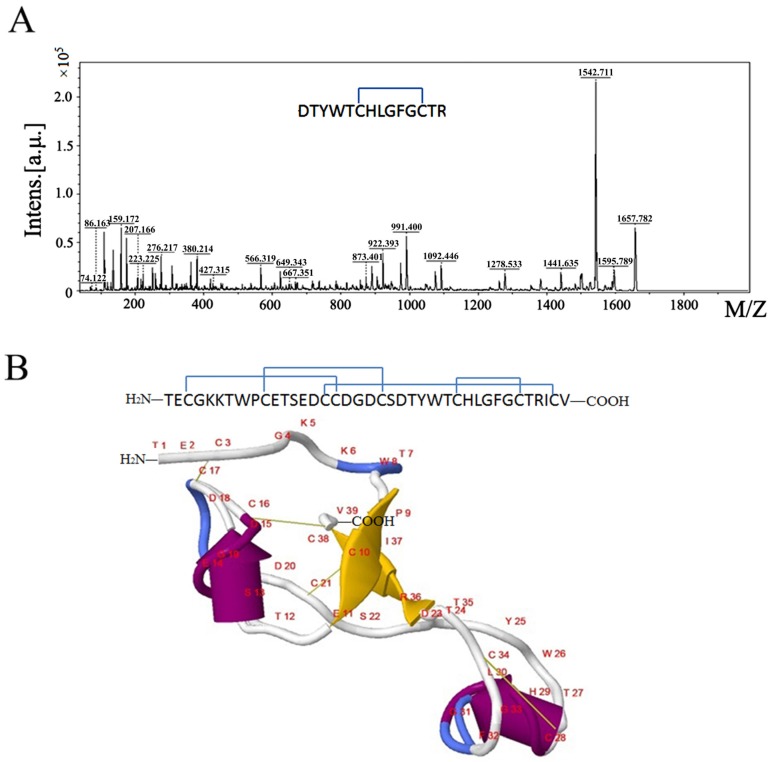
The MS spectra for mapping of the disulfide bond between Cys28 and Cys34 and the predicted secondary structure of µ-TRTX-Hl1a. (**A**) The MS/MS spectrum of peptide DTYWTCHLGFGCTR; and (**B**) the secondary structure and disulfide bonds of µ-TRTX-Hl1a predicted by Phyre 2. In the figure, the disulfide bonds are indicated in yellow line, alpha helices were shown as “yellow rockets”, beta strands were shown as “purple planks”; arrowheads point towards the carboxyl termini, random coils were colored in white, and turns were colored in blue.

The peptide sequence of µ-TRTX-Hl1a was searched against the PDB protein structure database. It matched well with insecticidal spider-venom peptide Aps III (PDB id: 2M36) with high confidence (92.64%) and 35 amino acid residues were identical to each other, including the eight Cys residues. The disulfide bonds of µ-TRTX-Hl1a were determined by on-line secondary structure modeling (on-line Phyre 2 tool [17], combined with protease digestion followed by MALDI-TOF-TOF-MSMS characterization. The insecticidal spider-venom peptide Aps III has 38 amino acid residues containing eight Cys residues forming four disulfide bonds. However, in the predicted secondary structure model, only three disulfide bonds (Cys3–Cys17, Cys10–Cys21, and Cys16–Cys38) were predicted with high confidence. Therefore, we used multiple protease digestions followed by MALDI-TOF/TOF-MSMS to analyze whether Cys28 and Cys34 form a disulfide bond. The peptide was digested with Trypsin/Lys-C Mix and Asp-N and a 14-amino-acid-residue peptide (DTYWTCHLGFGCTR) containing Cys 28 and Cys34 was generated and identified exhibiting a molecular mass of 1657.78 by MS/MS (Figure 3A). Its theoretical molecular mass was 2 Da higher than the detected one, which indicated that there was a disulfide bond between Cys28 and Cys34. In summary, four disulfide bonds were identified and paired in µ-TRTX-Hl1a as Cys3–Cys17, Cys10–Cys21, Cys16–Cys38, and Cys28–Cys34. The predicted secondary structure and disulfide bonds of µ-TRTX-Hl1a were presented with JSmolviewer (Figure 3B).

### 2.4. Effects of µ-TRTX-Hl1a on VGSCs

**Figure 4:**
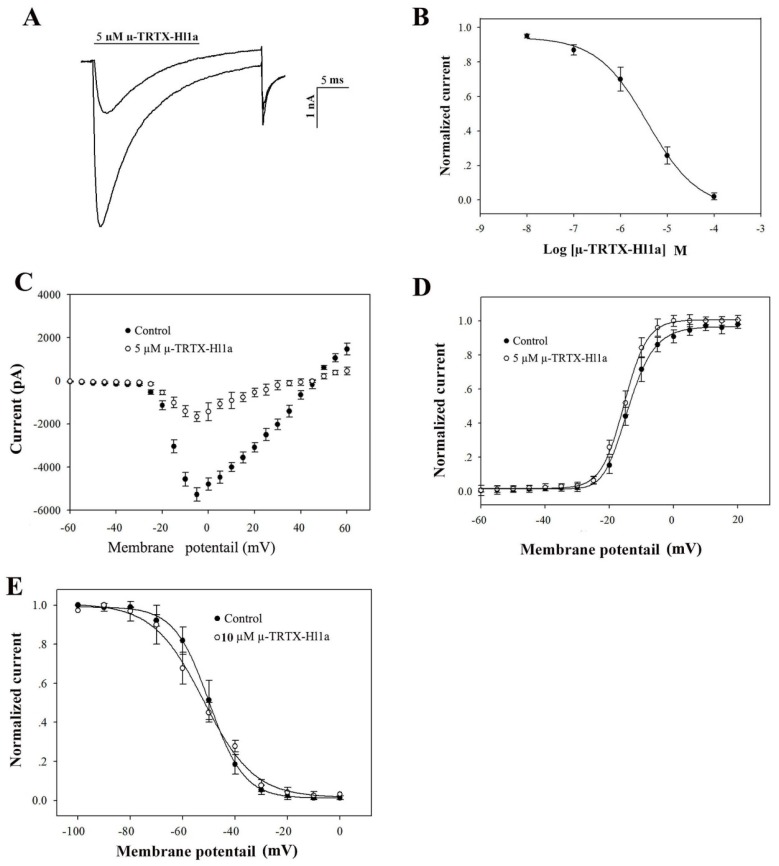
Effect of µ-TRTX-Hl1a on voltage-gated ion channels in rat DRG neurons. (**A**) Inhibition of TTX-r Na_V_ channel currents by 5 µM µ-TRTX-Hl1a; (**B**) dose-dependent inhibition of µ-TRTX-Hl1a on TTX-r Na_V_ channel currents (*n* = 5); (**C**) current-voltage (I-V) relationship for the TTX-r Na_V_ channel currents before (solid circles) and after (open circles) application of 5 µM µ-TRTX-Hl1a; (**D**) conductance-voltage (G-V) relationship for the TTX-r Na_V_ channel before (solid circles) and after (open circles) treatment of 5 μM µ-TRTX-Hl1a (*n* = 5); and (**E**) steady-state inactivation of the TTX-r Na_V_ channel currents before (solid circles) and after (open circles) application of 10 μM µ-TRTX-Hl1a (*n* = 5).

The investigation of the biological function of µ-TRTX-Hl1a was performed on adult rat DRG neurons by using the whole-cell patch-clamp technique. Cells were held at −80 mV for over 5 min to allow adequate equilibration. Currents were elicited by a 50 ms depolarizing potential of −10 mV from a holding potential of −80 mV every 5 s [18]. However, as shown in Figure 4A, about 72% of TTX-resistant (TTX-r) currents were inhibited by 5 µM µ-TRTX-Hl1a and the inhibition was dose-dependent with an IC_50_ value of 3.76 ± 0.10 μM (Figure 4B).

The current-voltage (I-V) curve of TTX-r sodium channel showed that the toxin did not alter the initial activated voltage and the reversal potentials of sodium channels, implying that the interaction between µ-TRTX-Hl1a and the sodium channel did not change ion selectivity (Figure 4C). In the presence of 5 μM µ-TRTX-Hl1a, conduction-voltage (G-V) relationship of sodium channel did not change. The estimated midpoint values of conductance-voltage relationship before and after toxin treatment were −5.20 and −8.6 mV, respectively (Figure 4D). The effects of µ-TRTX-Hl1a on steady-state inactivation of TTX-r sodium channel of DRG neuron were illustrated in Figure 4E. No shift of the steady-state inactivation curve of TTX-r sodium channel was induced by this toxin (Figure 4E).

### 2.5. Effects of µ-TRTX-Hl1a on Na_V_1.8

**Figure 5:**
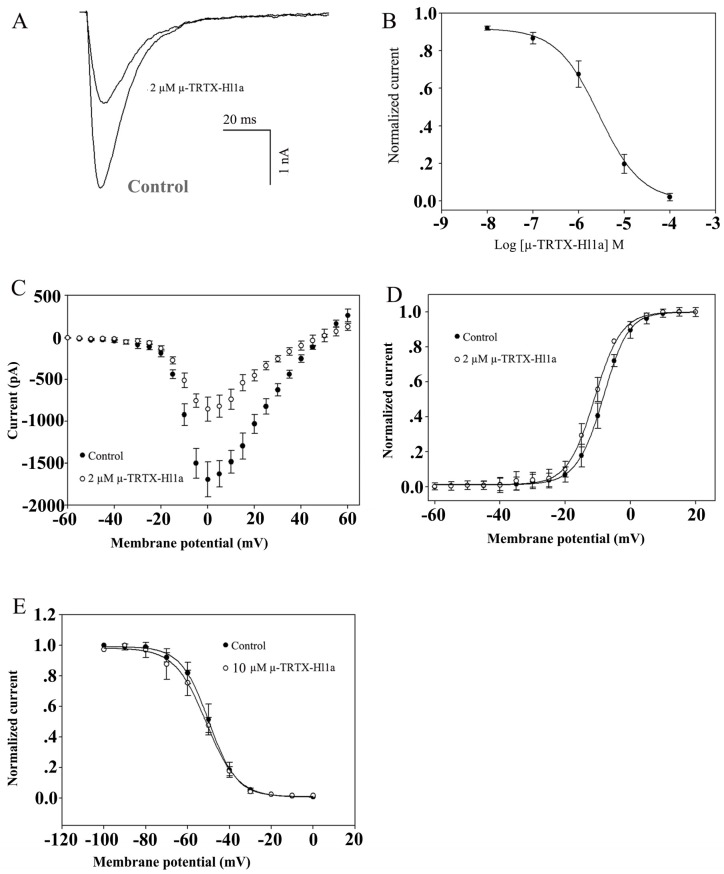
Effect of µ-TRTX-Hl1a on Nav1.8 expressed in ND7/23 cells; (**A**) current traces were evoked by a 50-ms step depolarization to −10 mV from a holding potential of −80 mV every 5 s. The effect of 2 μM µ-TRTX-Hl1a on the currents of Na_V_1.8; (**B**) dose-dependent inhibition of µ-TRTX-Hl1a on Na_V_1.8 channel; (**C**) the current-voltage (I-V) relationship for Na_V_1.8 channel currents before (solid circles) and after (open circles) application of 2 µM µ-TRTX-Hl1a; (**D**) the conductance-voltage (G-V) relationship for Na_V_1.8 channel before (solid circles) and after (open circles) treatment of 2 μM µ-TRTX-Hl1a (*n* = 5); and (**E**) steady-state inactivation of Na_V_1.8 channel currents before (solid circles) and after (open circles) application of 10 μM µ-TRTX-Hl1a (*n* = 5).

We have demonstrated that µ-TRTX-Hl1a inhibited TTX-r sodium currents in adult rat DRG neurons, but showed no effect on TTX-sensitive sodium currents at the concentration of 10 μM. Therefore, the activities of µ-TRTX-Hl1a on human sodium channel subtypes Na_V_1.1–Na_V_1.8 were further investigated. Sodium channel subtypes β1 and eGFP were transiently transfected into ND7/23 cells and currents were elicited by a depolarizing potential of −10 mV from a holding potential of −80 mV. 2 µM µ-TRTX-Hl1a could reduce about 50% of the Na_V_1.8 current and the inhibition was dose-dependent (Figure 5A). The IC_50_ value of µ-TRTX-Hl1a on Na_V_1.8 was 2.19 ± 0.07 μM (Figure 5B). Furthermore, no inhibition was found in the other subtypes (Nav1.1–1.7) of sodium channel (Figure S1). µ-TRTX-Hl1a at a concentration of 2 μM did not induce the shift of current-voltage (I-V) relationship and conductance-voltage (G-V) relationships of sodium channel subtype Na_V_1.8 (Figure 5C,D). As shown in Figure 5E, 10 μM µ-TRTX-Hl1a did not change the steady-state inactivation curve of Na_V_1.8.

### 2.6. Effects of µ-TRTX-Hl1a on Pain

Since Na_V_1.8 is a significant analgesic drug target, the analgesic effect of µ-TRTX-Hl1a was tested in several animal models including formalin-induced paw licking, tail-flicking, acetic acid-induced writhing, and a hot plate test.

**Figure 6:**
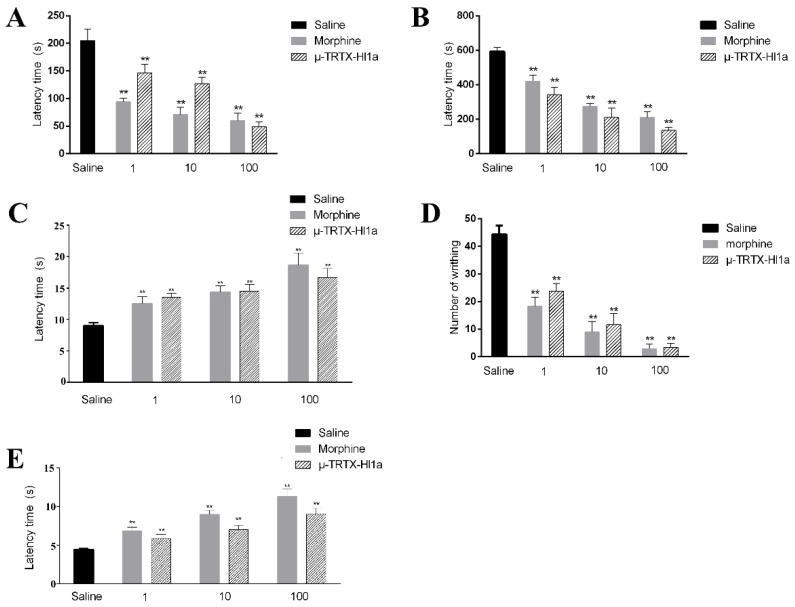
Effects of µ-TRTX-Hl1a on pain. (**A**) Effects of µ-TRTX-Hl1a on the early phase of formalin-induced paw licking response in mice; (**B**) effects of µ-TRTX-Hl1a on the late phase of formalin-induced paw licking response in mice; (**C**) eEffects of µ-TRTX-Hl1a on the hot plate test in mice; (**D**) effects of µ-TRTX-Hl1a on the acetic acid-induced writhing response in mice; and (**E**) effects of µ-TRTX-Hl1a on tail flicking test in mice. All data points are shown as mean ± *S.E*. Six animals were used for each separate group experiment. * *p <* 0.05, ** *p <* 0.01 significantly different results compared to the saline group.

Two time phases, including the early phase (0–15 min, neurogenic pain) and the late phase (15–30 min, inflammatory pain), were recorded in response to formalin-induced paw licking. In the control mice group, paw licking time was 205 s in the early phase and 592 s in the late phase (Figure 6A,B). In the early phase, the paw licking times were 146, 127, and 49 s for 1, 10, and 100 µM/kg µ-TRTX-Hl1a, respectively, while the numbers were 94, 71, and 60 s after injection of the same concentrations of morphine (Figure 6A). In the late phase, the paw licking times were reduced to 341, 210, and 136 s for mice treated by 1, 10, and 100 µM/kg µ-TRTX-Hl1a, respectively, while the values were decreased to 421, 275, and 211 s after injection of the same concentrations of morphine (Figure 6B). In the hot plate test, a strong analgesic effect was observed after administration of µ-TRTX-Hl1a or morphine. Compared with the latency of control (9 s), the latencies of mice treated by 1, 10, and 100 µM/kg µ-TRTX-Hl1a was 13.5, 14.5, and 16.4 s, respectively. The latencies of 1, 10, and 100 µM/kg morphine were 12.5, 14.3, and 18.6 s, respectively (Figure 6C). In the acetic acid-induced writhing test, intraperitoneal injection of 1, 10, and 100 µM/kg µ-TRTX-Hl1a reduced the duration of writhing from 44.3 to 23.8, 11, and 3.5, respectively, while injection of 1, 10, and 100 µM/kg morphine caused a reduction to 18, 9, and 3, respectively (Figure 6D). As illustrated in Figure 6E, the tail withdrawal latency was 4.4 s in the control group. After treatment with 1, 10, and 100 µM /kg µ-TRTX-Hl1a, the latency of mice was 5.8, 7, and 9 s, respectively. Meanwhile, the latencies of mice treated with the same concentrations of morphine were 6.8, 8.9, and 11.3 s, respectively.

## 3. Discussion

Animal venoms are a rich source of peptides and thousands of peptide toxins have been found in venoms. Some peptides have shown high affinity and selectivity for a diverse range of biological targets, especially membrane proteins, such as ion channels, receptors, and transporters [2,19]. Six drugs derived from venom peptides or proteins (captopril, eptifibatide, ziconotide, exenatide, tirofiban, and bivalirudin) have been approved by the Food and Drug Administration [20]. Therefore, peptide toxins may be a new library for modern drug discovery. *H. lividum*, as one tarantula species of spiders in Southeast Asia, has never been intensively investigated so far. Only glutamic acid, histamine, and adenosine were identified from the venom of *H. lividum*, while no peptide toxin has been reported [21].

In this study, we described the identification and functional characterization of a novel neurotoxin µ-TRTX-Hl1a from the spider venom of *H. lividum.* It consists of 39 residues and four disulfide bonds. µ-TRTX-Hl1a showed identity to some other spider toxins such as 72% to JZTX-59 (U29-TRTX-Cj1a), 45% to toxic peptide C (U1-NETX-Csp1a) and 39% to Aps III (μ-cyrtautoxin-As1a). Although JZTX-59 was considered to be a neurotoxin, its target is still unknown. Other two toxins, toxic peptide C (U1-NETX-Csp1a) and Aps III (μ-cyrtautoxin-As1a), are insecticidal peptides.

Linear µ-TRTX-Hl1a was synthesized and different solutions were used to refold this peptide. Technically, it is very hard to refold a peptide toxin with more than three disulfide bonds. In this work, we found that the condition of low pH (6.8) and low temperature (4 °C) were the optimum conditions for refolding of µ-TRTX-Hl1a. A variety of chemical additives, e.g., urea, guanidine hydrochloride (Gu/HCl), and arginine, could improve refolding of disulfide-coupled proteins [22]. The refolding was increased from 8.52% to 10.38% with the addition of 0.5 M *L*-Arg, but greatly increased the complexity of refolded protein. Finally, half of the concentration of the salt (0.05 M Tris-HCl, 0.05 M NaCl, 0.15 mM GSSG, 1.5 mM GSH) was applied and the percentage was increased from 8.52% to 13.46%. Thus, low pH, low temperature, and low salt concentration may be helpful for refolding of peptides with many disulfide bonds.

The cysteine pattern of µ-TRTX-Hl1a was similar to ApsIII, a toxin containing an atypical ICK motif with a conserved cysteine pattern [16], indicating that they may adopt the same motif. Structure modeling implied that three disulfide bonds of µ-TRTX-Hl1a matched with Aps III, but the other bond (Cys 28 and Cys34) was uncertain. This cysteine framework was further determined by protease digestion and MS identification, implying that µ-TRTX-Hl1a is an ICK motif-containing toxin. Taken together, MS combined with structure prediction is an efficient way to determine the disulfide bonds of a long-chain peptide.

Clinical genetic studies have demonstrated that sodium channel subtype Na_V_1.8 (SCN9A) is a critical mediator of pain sensitization and it becomes an attractive target for the development of novel analgesics [9]. Various commercially available compounds, such as menthol, lidocaine, tetracaine, vinpocetine, ambroxol, lamotrigine, mexiletine, veratridine, and A-803467, interact with Na_V_1.8, whereas very few animal toxins have been shown to reduce Na_V_1.8 currents. Recent studies revealed that tarantula toxins (ProTx-I, ProTx-II, CcoTx1, CcoTx2, and CcoTx3) could inhibit rat Na_V_1.8 currents, but none selectively targeted Na_V_1.8 [23,24]. µ-TRTX-Hl1a did not alter the I-V relationship, conduction-relationship, and steady-state inactivation. The mechanism of inhibition was very similar to other spider toxins [25,26]. µ-TRTX-Hl1a selectively reduced the current amplitude of Na_V_1.8 with an IC_50_ value of 2.19 μM without affecting other sodium subtypes (Na_V_1.1, Na_V_1.2, Na_V_1.4, Na_V_1.5, Na_V_1.6, and Na_V_1.7). We did not find any effect of µ-TRTX-Hl1a on potassium channels and calcium channels (Figure S2). Due to its highly selective activity on Na_V_1.8, µ-TRTX-Hl1a could be a valuable tool for the study of structure-function of Na_V_1.8.

As a highly selective inhibitor of Na_V_1.8, µ-TRTX-Hl1a may have analgesic effects. To confirm this hypothesis, several experimental pain models, including formalin paw licking, tail flicking, hot plate test, and abdominal writhing were used to analyze the analgesic effects of µ-TRTX-Hl1a. There was a significant decrease of nociception in all the rodent models with almost the same efficacy to morphine at the molar basis. In the formalin-induced pain model, µ-TRTX-Hl1a showed an equipotent or less analgesic effect to morphine in the early phase, whereas a higher analgesic effect than morphine was observed in the later phase (Figure 6A,B). µ-TRTX-Hl1a was slightly less effective than morphine in the tail-flicking test and hot plate test, but showed a better effect in an acetic acid-induced writhing test (Figure 6C–E). This is consistent with the previous reports that inhibition of Na_V_1.8 elicited analgesic effects in neuropathic and inflammatory pain. In our study, µ-TRTX-Hl1a was more effective on inflammatory pain than on neuropathic pain, implying Na_V_1.8 may have different effects on the two different kinds of pain.

This study intensively characterized the secondary structure and biological activity of µ-TRTX-Hl1a, which is the first neurotoxin identified from the venom of *H. lividum.* Its highly selective activity for Na_V_1.8 indicates that µ-TRTX-Hl1a is a potential tool for the study of the physiology of Na_V_1.8. At the same time, it could be a lead compound or template for the development of novel analgesic agents.

## 4. Materials and Methods

### 4.1. cDNA Synthesis

Total RNA was extracted from the venom glands of the spider using TRIzol (Thermo Fisher Scientific, Waltham, MA, USA) and checked by visualizing the 28S, 18S, and 5S bands of ribosomal RNA. cDNA library was constructed by using a SMART™ PCR cDNA synthesis kit (Clontech, Palo Alto, CA, USA) [14]. The first strand was synthesized by using the 3′ SMART CDS Primer II A (5′ AAGCAGTGGTATCAACGCAGAGTACT(30)N-1N 3′, where N = A, C, G, or T and N-1 = A, G, or C) and SMART II A oligonucleotide (5′ AAGCAGTGGTATCAACGCAGAGTACGCGGG 3′). The 5′ PCR primer II A (5′ AAGCAGTGGTATCAACGCAGAGT 3′) provided by the kit was used to synthesize the second strand using Advantage Polymerase (Clontech, Palo Alto, CA, USA).

### 4.2. cDNA Sequence and Bioinformatics Analysis

PCR was performed with the M13 forward and reverse primers in the kit to rapidly screen recombinant clones. The clones from original cDNA library were separated on LB agar plates. The resulting colonies were randomly picked and sequenced with standard M13 reverse primers on an ABI 3730 (ABI, Waltham, MA, USA) automatic DNA sequencer according to the manufacturer’s instructions. The sequences of ESTs were translated into amino acid sequences following the forward three frames using the software Jemboss 1.3 (EMBOSS, London, UK). The signal peptide was predicted with the SignalP 4.1 program [27].

### 4.3. Peptide Synthesis and Refolding

The peptide predicted based on the sequences of ESTs was synthesized with solid-phase methodology using a Boc protection strategy in GL Biochem Ltd. (GL Biochem, Shanghai, China). The linear peptides (0.1 mg) were dissolved in 1 mL different renaturation solutions of different pH (Table 1). 10 μL 50% TFA was used to terminate the reaction. After oxidization and refolding, the oxidized peptide was isolated by analytical RP-HPLC on the C_18_ preparative column at 280 nm.

### 4.4. Mass Spectrometric Analysis

The purified peptide was dissolved in 0.1% (*v*/*v*) trifluoroacetic acid and 0.5 µL was spotted onto a matrix-assisted laser desorption ionization time-of-flight (MALDI-TOF) plate with 0.5 µL α-cyano-4-hydroxycinnamic acid matrix (10 mg/mL in 60% acetonitrile). Spots were analyzed by an UltraFlex I mass spectrometer (Bruker Daltonics, Bremen, Germany) in a positive ion mode to determine the molecular mass of the linear and refolded peptides according to manufacturer’s instruction.

### 4.5. Digestion of the Peptide

Multiple proteases were used to generate peptide fragments with disulfide bond. In the experiment, 50 μg of refolded µ-TRTX-Hl1a was dissolved in 100 μL of l50 mM triethylammonium bicarbonate and firstly digested with Trypsin/Lys-C Mix (Promega, Copenhagen, Denmark) (2%, *w*/*w*) at 37 °C overnight. The tryptic peptide was then subjected to AspN (Promega, Copenhagen, Denmark) digestion (4%, *w*/*w*) at 37 °C for 15 h.

### 4.6. MALDI-TOF/TOF-MSMS

The purified peptide and digested peptide fragments after each protease treatment were desalted by reversed phase chromatography using Poros Oligo R3 reversed-phase material staged in GE Loader tips. Retained peptides were eluted onto a stainless-steel target plate using 70% ACN in 1% TFA containing 5 mg/mL α-CHCA. Mass spectra were acquired on an Ultraflex II MALDI–TOF/TOF (tandem TOF) mass spectrometer controlled by flexControl software (version 2.4, Bruker Daltonics, Bremen, Germany). The instrument was operated in the positive reflector ion detection mode, and spectra were recorded in the mass range of *m*/*z* 500–4000. Typically, signals from 1000 laser shots (10 × 100 shots at 10 different positions) were averaged. Spectra were externally calibrated using a tryptic lactoglobulin. Mass spectra were analyzed with Flex-Analysis software (version 2.4, Bruker Daltonics, Bremen, Germany). For MALDI–TOF/TOF analysis, the window for precursor ion selection was set to be ±1% of the mass of the precursor ion.

### 4.7. Structure Prediction and Modeling

The structure was predicted and modeled using the on-line Phyre 2 tool [17] and JSmolviewer following the instructions.

### 4.8. Patch Clamp Recording on Rat Dorsal Root Ganglion (DRG) Neurons

Thirty-day-old adult Sprague-Dawley rats of either sex were decapitated. Then the dorsal root ganglia were isolated quickly from the spinal cord [20]. The dissociated cells were suspended in essential Dulbecco’s modified Eagle’s medium containing trypsin (0.5 g/L, type III), collagenase (1.0 g/L, type IA), and DNase (0.1 g/L, type III) to incubate at 34uC for 30 min. Trypsin inhibitor (1.5 g/L, type II-S) was used to terminate enzyme treatment. The DRG cells were transferred into a 35-mm culture dish. Rat DRG neurons were acutely dissociated and maintained in a short-term primary culture. Ca^2+^, Na^+^, and K^+^ currents were recorded from cells using the whole-cell patch clamp technique with an Axon Multiclamp 700B amplifier (Molecular Devices, CA, USA). The P/4 protocol was used to subtract linear capacitive and leakage currents. For sodium current recordings on DRG cells, the bath solution contained (in mM): 150 NaCl, 2 KCl, 5 D-glucose, 1 MgCl_2_, 1.5 CaCl_2_, and 10 HEPES at pH 7.4; the pipette internal solution contained (in mM): 105 CsF, 35 NaCl, 10 HEPES, and 10 EGTA at pH 7.4. For potassium channel, pipettes were filled with a solution containing (in mM) 76 K_2_SO4, 10 KCl, 10 NaCl, 1 MgCl_2_ and 5 HEPES, pH adjusted to 7.4, with KOH. The external solution was (in mmol/l) 140 NaCl, 3.6 KCl, 2 NaHCO_3_, 0.5 NaH_2_PO_4_, 2.6 CaCl_2_, 0.5 MgSO_4_, and 5 HEPES, pH 7.4. For the calcium channel, the standard intracellular solution contained (in mM): 135 CsCl; 3 MgCl_2_; 10 EGTA; 10 HEPES; 3 Mg-ATP; 0.6 GTP (pH 7.4 adjusted with CsOH). The standard bath solution contained (in mM): 150 TEA-Cl; 2 CaCl_2_; 10 HEPES; 10 glucose (pH 7.4 adjusted with CsOH). LVA currents were measured by 50 ms square voltage pulses to −40 mV from a holding potential of −90 mV. Recordings were sampled at 4 kHz.

The patch pipettes with DC resistances of 2–3 M were fabricated from borosilicate glass tubing (VWR micropipettes, 100 mL, VWR, Radnor, PA, USA) using a two-stage vertical microelectrode puller (PC-10, Narishige, Tokyo, Japan) and fire-polished by a heater (Narishige, Tokyo, Japan). All of the experimental protocols using animals in this work were approved by the Animal Care and Use Committee of Kunming Institute of Zoology, Chinese Academy of Sciences (2014-204, 14 October 2014).

### 4.9. Patch Clamp Recording on Sodium Channel Subtypes

Human sodium channel subtype Nav1.1–Nav1.7 were transiently transfected into HEK293T cells and Na_V_1.8, β1, and eGFP were transiently transfected into ND7/23cells following the manufacture instruction (lipo2000, Invitrogen, Carlsbad, CA, USA). The whole cell patch clamp recordings were carried out as previously described [28]. The standard pipette solution contained (in mM) 140 CsF, 1 EGTA, 10 NaCl, 3 KCl, and 10 MgCl_2_, PH 7.3. The standard bathing solution was (in mM) 140 NaCl, 3 KCl, 1 MgCl_2_, 1 CaCl_2_, and 10 HEPES, pH 7.3.

### 4.10. Formalin-Induced Paw Licking

A formalin test was performed according to the method described by Owoyele et al. [29]. Mice were intraperitoneally (i.p.) injected with test samples dissolved in 100 μL of saline and positive control animals were intraperitoneally injected with morphine dissolved in saline. The control group received the same volume of saline. Animals were injected with 20 μL of 5% formalin at the plantar surface of right hind paw after intraperitoneally injected with test samples. They were then placed into open polyvinyl cages (20 cm × 40 cm × 15 cm) individually. The time spent for licking the injected paw by each mouse was recorded by a digital video camera (early phase or neurogenic pain, 0–15 min post-injection and late phase or inflammatory pain, 15–30 min post-injection).

### 4.11. Tail-Flicking Test

Mice with pain threshold of 4–6 s were selected for the tail-flicking test. Test samples (morphine and µ-TRTX-Hl1a) were dissolved in saline. A photothermal pain detector (YLS-12A, Jinan, Shandong, China) was used to measure the pain threshold of mice subjected to intense heat and the light beam of the detector was focused on the middle of mouse tail. All of the animals were intraperitoneally injected with saline containing different concentrations of samples and control group received the same volume of saline [30]. Tail withdrawal latency was measured as the time taken to withdraw the tail from the light beam.

### 4.12. Abdominal Writhing Response Caused by Acetic Acid

According to the method described by Santos et al. [31], mice were immediately intraperitoneally injected with saline containing different concentrations of samples after intraperitoneal injection of 200 μL of 0.8% (*v*/*v*) acetic acid. The control group received the same volume of saline. After challenging, mice were placed into open polyvinyl cages (20 × 40 × 15 cm) individually, and the abdominal writhing responses were counted for 30 min continuously.

### 4.13. Hot Plate

A hot plate apparatus (model HZ66-ZH-YLS-6B, Shanghai, China), maintained at 55 ± 1 °C, was used to test the pain in response to a thermal stimulus [32]. Mice in the control condition showing initial nociceptive responses between 5 and 10 s were selected for the experiment. The latency to the sign of hind paw licking or jumping to avoid heat nociception was taken as the index of the nociceptive threshold. Measurements were immediately started after treatment of the animals with µ-TRTX-Hl1a or the vehicle. A group of mice treated with morphine dissolved in physiological saline was used as a positive control. The vehicle used alone had no effect on nociceptive responses.

### 4.14. Statistics

#### 4.14.1. Data analysis for Patch Clamp 

Experiments data were acquired and analyzed by using Clampfit 10.0 (Molecular Devices, Sunnyvale, CA, USA) and Sigmaplot (Systat Software Incx, San Jose, CA, USA) programs. Data were analyzed using the Clampfit (Axon, Sunnyvale, CA, USA) and Sigmaplot 9.0 (Systat Software Incx, San Jose, CA, USA) software programs. All data points are shown as mean ± *S.E*. *n* represents the number of the separate experimental cells. Dose-response curves were fitted using the following Hill logistic equation: y = 1 − (1 − f_max_)/(1 − ([Tx]/IC^50^)^n^), where n is an empirical Hill coefficient and fmax is the fraction of current resistant to inhibition at high toxin concentration (Tx). Conductance (G)–voltage relationships were determined from peak current (I) versus voltage relationships as G = I/(V − Vrev), where V was the test potential, and Vrev was the extrapolated reversal potential. Data were analyzed by using GraphPad Prism 4.

#### 4.14.2. Data Analysis of the Animal Model

The results are expressed as the mean ± standard error of the mean (*SEM*). Statistical significance was calculated using one- or two-way analysis of variance followed by the Student-Newman-Keuls and Bonferroni’s post hoc tests. Significance was defined as *p* < 0.05.

## Figures and Tables

**Table 1:** Refold of µ-TRTX-Hl1a at different condition.

Buffer condition	Temperature (℃)	Duration (h)	pH	Folding Yield %
0.1 mol/L Tris-HCl, 0.1 mol/L NaCl, 0.3 mmol/L GSSG, 3 mmol/L GSH	25	24	7.2	6.04
	4	24	7.2	7.08
	4	24	6.8	8.52
	4	48	6.8	8.46
0.1 mol/L Tris-HCl, 0.1 mol/L NaCl, 0.3 mmol/L GSSG, 3 mmol/L GSH, 1 mol/L L-Arg	4	24	7.6	8.13
	4	24	7.4	8.53
	4	24	7.2	9.39
	4	24	6.8	10.38
0.05 mol/L Tris-HCl, 0.05 mol/L NaCl, 0.15 mmol/L GSSG, 1.5 mmol/L GSH	4	24	7.2	10.20
	4	24	6.8	13.46

## Supplementary Materials

The following are available online at www.mdpi.com/2072-6651/9/1/7/s1, Figure S1 µ-TRTX-Hl1a showed no effect on Nav1.1, Nav1.2, Nav1.3, Nav1.4, Nav1.5, Nav1.6 and Nav1.7. Figure S2 µ-TRTX-Hl1a showed no effect on K_V_ (A) currents and Ca_V_ (B) currents of DRG neurons.

## References

[1] Herzig V., Wood D.L., Newell F., Chaumeil P.A., Kaas Q., Binford G.J., Nicholson G.M., Gorse D., King G.F. (2011). Arachnoserver 2.0, an updated online resource for spider toxin sequences and structures. Nucleic Acids Res..

[2] Klint J.K., Senff S., Rupasinghe D.B., Er S.Y., Herzig V., Nicholson G.M., King G.F. (2012). Spider-venom peptides that target voltage-gated sodium channels: Pharmacological tools and potential therapeutic leads. Toxicon.

[3] Takaoka M., Nakajima S., Sakae H., Nakamura T., Tohma Y., Shiono S., Tabuse H. (2001). Tarantulas bite: Two case reports of finger bite from *Haplopelma lividum*. Chudoku Kenkyu.

[4] Gaskin D.J., Richard P. (2012). The economic costs of pain in the united states. J. Pain.

[5] Spencer C.I. (2009). Actions of atx-ii and other gating-modifiers on Na(+) currents in HEK-293 cells expressing WT and deltaKPQ hNa(v) 1.5 Na(+) channels. Toxicon.

[6] Catterall W.A., Perez-Reyes E., Snutch T.P., Striessnig J. (2005). International union of pharmacology. Xlviii. Nomenclature and structure-function relationships of voltage-gated calcium channels. Pharmacol. Rev..

[7] Payandeh J., Scheuer T., Zheng N., Catterall W.A. (2011). The crystal structure of a voltage-gated sodium channel. Nature.

[8] Shields S.D., Ahn H.S., Yang Y., Han C., Seal R.P., Wood J.N., Waxman S.G., Dib-Hajj S.D. (2012). Nav1.8 expression is not restricted to nociceptors in mouse peripheral nervous system. Pain.

[9] Faber C.G., Lauria G., Merkies I.S., Cheng X., Han C., Ahn H.S., Persson A.K., Hoeijmakers J.G., Gerrits M.M., Pierro T. (2012). Gain-of-function nav1.8 mutations in painful neuropathy. Proc. Natl. Acad. Sci. USA.

[10] Garrison S.R., Weyer A.D., Barabas M.E., Beutler B.A., Stucky C.L. (2014). A gain-of-function voltage-gated sodium channel 1.8 mutation drives intense hyperexcitability of a- and c-fiber neurons. Pain.

[11] Bierhaus A., Fleming T., Stoyanov S., Leffler A., Babes A., Neacsu C., Sauer S.K., Eberhardt M., Schnolzer M., Lasitschka F. (2012). Methylglyoxal modification of nav1.8 facilitates nociceptive neuron firing and causes hyperalgesia in diabetic neuropathy. Nat. Med..

[12] Jarvis M.F., Honore P., Shieh C.C., Chapman M., Joshi S., Zhang X.F., Kort M., Carroll W., Marron B., Atkinson R. (2007). A-803467, a potent and selective nav1.8 sodium channel blocker, attenuates neuropathic and inflammatory pain in the rat. Proc. Natl. Acad. Sci. USA.

[13] King G.F., Gentz M.C., Escoubas P., Nicholson G.M. (2008). A rational nomenclature for naming peptide toxins from spiders and other venomous animals. Toxicon.

[14] Chen J., Deng M., He Q., Meng E., Jiang L., Liao Z., Rong M., Liang S. (2008). Molecular diversity and evolution of cystine knot toxins of the tarantula *Chilobrachys jingzhao*. Cell. Mol. Life Sci. CMLS.

[15] Skinner W.S., Dennis P.A., Li J.P., Quistad G.B. (1992). Identification of insecticidal peptides from venom of the trap-door spider, *Aptostichus schlingeri* (Ctenizidae). Toxicon.

[16] Bende N.S., Kang E., Herzig V., Bosmans F., Nicholson G.M., Mobli M., King G.F. (2013). The insecticidal neurotoxin aps iii is an atypical knottin peptide that potently blocks insect voltage-gated sodium channels. Biochem. Pharmacol..

[17] Phyre 2 tool. http://www.sbg.bio.ic.ac.uk/phyre2.

[18] Deng M., Kuang F., Sun Z., Tao H., Cai T., Zhong L., Chen Z., Xiao Y., Liang S. (2009). Jingzhaotoxin-ix, a novel gating modifier of both sodium and potassium channels from Chinese tarantula *Chilobrachys jingzhao*. Neuropharmacology.

[19] Saez N.J., Senff S., Jensen J.E., Er S.Y., Herzig V., Rash L.D., King G.F. (2010). Spider-venom peptides as therapeutics. Toxins.

[20] Yang S., Xiao Y., Kang D., Liu J., Li Y., Undheim E.A., Klint J.K., Rong M., Lai R., King G.F. (2013). Discovery of a selective Nav1.7 inhibitor from centipede venom with analgesic efficacy exceeding morphine in rodent pain models. Proc. Natl. Acad. Sci. USA.

[21] Moore S., Smyth W.F., Gault V.A., O’Kane E., McClean S. (2009). Mass spectrometric characterisation and quantitation of selected low molecular mass compounds from the venom of *Haplopelma lividum* (Theraphosidae). RCM.

[22] Okumura M., Shimamoto S., Hidaka Y. (2014). Chemical methods for producing disulfide bonds in peptides and proteins to study folding regulation. Curr. Protoc. Protein Sci..

[23] Middleton R.E., Warren V.A., Kraus R.L., Hwang J.C., Liu C.J., Dai G., Brochu R.M., Kohler M.G., Gao Y.D., Garsky V.M. (2002). Two tarantula peptides inhibit activation of multiple sodium channels. Biochemistry.

[24] Bosmans F., Rash L., Zhu S., Diochot S., Lazdunski M., Escoubas P., Tytgat J. (2006). Four novel tarantula toxins as selective modulators of voltage-gated sodium channel subtypes. Mol. Pharmacol..

[25] Deng M., Luo X., Jiang L., Chen H., Wang J., He H., Liang S. (2013). Synthesis and biological characterization of synthetic analogs of huwentoxin-iv (mu-theraphotoxin-hh2a), a neuronal tetrodotoxin-sensitive sodium channel inhibitor. Toxicon.

[26] Xiao Y., Blumenthal K., Cummins T.R. (2014). Gating-pore currents demonstrate selective and specific modulation of individual sodium channel voltage-sensors by biological toxins. Mol. Pharmacol..

[27] SignalP 4.1 program. http://www.cbs.dtu.dk/services/SignalP/.

[28] Rong M., Chen J., Tao H., Wu Y., Jiang P., Lu M., Su H., Chi Y., Cai T., Zhao L. (2011). Molecular basis of the tarantula toxin jingzhaotoxin-iii (beta-trtx-cj1alpha) interacting with voltage sensors in sodium channel subtype nav1.5. FASEB J..

[29] Owoyele V.B., Adediji J.O., Soladoye A.O. (2005). Anti-inflammatory activity of aqueous leaf extract of *Chromolaena odorata*. Inflammopharmacology.

[30] Wei L., Dong L., Zhao T., You D., Liu R., Liu H., Yang H., Lai R. (2011). Analgesic and anti-inflammatory effects of the amphibian neurotoxin, anntoxin. Biochimie.

[31] Santos J.A., Arruda A., Silva M.A., Cardoso C.A., Vieira Mdo C., Kassuya C.A., Arena A.C. (2012). Anti-inflammatory effects and acute toxicity of hydroethanolic extract of jacaranda decurrens roots in adult male rats. J. Ethnopharmacol..

[32] Zhu Y., Li Z., Liu H., He X., Zhang Y., Jin J., Che J., Li C., Chen W., Lai R. (2014). Novel analgesic peptides from the tree frog of *Hyla japonica*. Biochimie.

