# Supplementary material for: A Novel Toxin from Haplopelma lividum Selectively Inhibits the NaV1.8 Channel and Possesses Potent Analgesic Efficacy
Source: Toxins (Basel). 2016 Dec 26;9(1):7. doi: 10.3390/toxins9010007 (PMC5308240; doi:10.3390/toxins9010007)
Supplement: Supplementary file 1 [file toxins-09-00007-s001.pdf]

# Supplementary Materials: A Novel Toxin from *Haplopelma lividum* Selectively Inhibits Nav1.8 Channel and Possesses Potent Analgesic Efficacy

**Ping Meng, Honggang Huang, Gan Wang, Shilong Yang, Qiuming Lu, Jingze Liu, Ren Lai and Mingqiang Rong**

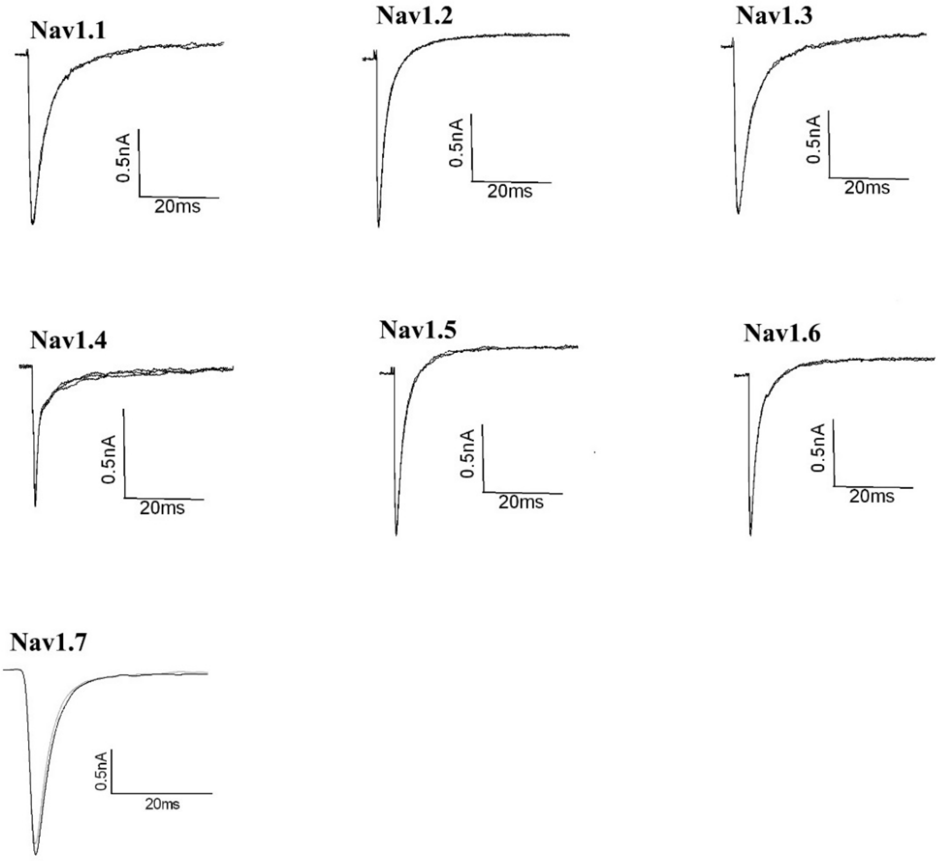


**Figure S1.** μ-TRTX-Hl1a showed no effect on Nav1.1, Nav1.2, Nav1.3, Nav1.4, Nav1.5, Nav1.6 and Nav1.7.

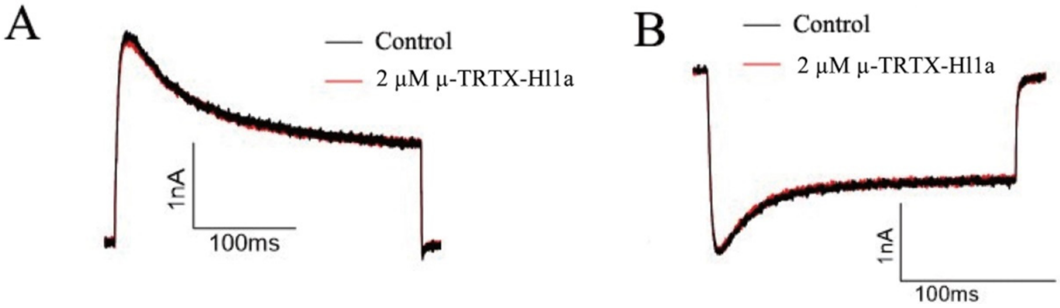


**Figure S2.** μ-TRTX-Hl1a showed no effect on Kv (**A**) currents and Cav (**B**) currents of DRG neurons.
